# A PTPRO-Related Five-Gene Blood Transcriptional Signature with Diagnostic Potential for Tuberculosis

**DOI:** 10.3390/biomedicines14051021

**Published:** 2026-04-30

**Authors:** Fengjiao Wu, Ru Huang, Yuxuan Lin, Xixi Zhu, Yujie Li, Huiting Dai, Xiaoyu Zhou, Fang Fang, Ying Liang, Tao Xu, Chuanwang Song, Wei Li, Xiaojing Wang, Xianyou Chang, Hongtao Wang, Ting Wang, Jingzhu Lv, Zhongqing Qian

**Affiliations:** 1 Anhui Provincial Key Laboratory of Immunology in Chronic Diseases, Anhui Provincial Key Laboratory of Infection and Immunology, Anhui Province Key Laboratory of Basic and Translational Research of Inflammation-related Diseases, Department of Immunology, Interdisciplinary Eye Research Institute (EYE-X Institute), College of Laboratory Medicine, Bengbu Medical University, Bengbu 233030, China; 2 Anhui Clinical and Preclinical Key Laboratory of Respiratory Disease, Department of Respiration, First Affiliated Hospital, Bengbu Medical University, Bengbu 233000, China; 3 The Infectious Disease Hospital of Bengbu City, Bengbu 233000, China; 4 Center for Translational Science, Florida International University, 11350 SW Village Parkway, Port St. Lucie, FL 34987, USA; 5 Key Laboratory of Industrial Dust Deep Reduction and Occupational Health and Safety of Anhui Higher Education Institutes, Huainan 232001, China

**Keywords:** *Mycobacterium tuberculosis*, PTPRO, tuberculosis, blood transcriptional signature, biomarker candidate

## Abstract

**Background/Objectives:** Tuberculosis (TB), caused by *Mycobacterium tuberculosis* (MTB), remains a major global health problem. Drug resistance and the limitations of sputum-based diagnostic methods highlight the need for additional host-response biomarkers. Protein tyrosine phosphatase receptor type O (PTPRO) has been implicated in inflammatory signaling and macrophage immune regulation, but its relationship with TB-related host transcriptional responses remains unclear. This study aimed to identify and preliminarily evaluate a PTPRO-related blood transcriptional signature with potential relevance to TB discrimination and treatment-response assessment. **Methods:** Genes correlated with PTPRO expression were first screened using TCGA-LUSC as a large human transcriptomic discovery resource. The resulting candidate genes were then filtered in TB-related whole-blood datasets by intersecting genes upregulated in TB compared with healthy controls, pneumonia, and lung cancer. This strategy yielded a five-gene PTPRO-related signature, termed PO5. The signature was evaluated in independent GEO cohorts and further explored by RT-qPCR in H37Ra-infected THP-1-derived macrophages and in a small clinical blood cohort. A PO5-derived TB risk score was calculated for each sample, and receiver operating characteristic analysis was used to assess discriminatory performance. Changes in TB risk scores during anti-TB treatment were also examined. **Results:** PTPRO expression was increased in TB whole-blood transcriptomic data and in H37Ra-infected macrophages. In public datasets, PO5 showed potential for distinguishing TB from healthy controls, latent TB, pneumonia, and lung cancer. PO5-derived TB risk scores also decreased after anti-TB treatment. In the exploratory clinical cohort, several PO5 genes showed expression changes in the same general direction as those observed in the public datasets, although the small sample size limited the strength of this evidence. **Conclusions:** PO5 represents a preliminary PTPRO-related blood transcriptional signature with potential relevance to TB discrimination and treatment-response assessment. These findings remain exploratory and require validation in larger prospective multicenter cohorts, together with further mechanistic studies.

## 1. Introduction

*Mycobacterium tuberculosis* (MTB) serves as the principal causative agent of the *Mycobacterium tuberculosis* complex, responsible for tuberculosis (TB), historically known as the “white plague” due to its high prevalence and mortality. In recent years, the global burden of TB has intensified with the rise of AIDS, the rise of multidrug-resistant (MDR), and extensively drug-resistant (XDR) MTB strains. The widespread use of immunosuppressive therapies has further increased susceptibility to TB, making tuberculosis a major public health challenge worldwide [1]. As stated by the World Health Organization (WHO), in 2022 there were 10.6 million new tuberculosis cases globally, with 1.3 million associated deaths. Of these, 630,000 (6.3%) had HIV co-infection, and 410,000 (3.9%) were multidrug/rifampicin-resistant cases. Resistance was detected in 3.3% of new patients and 17% of relapsed patients [2]. The widespread burden of TB and the emergence of drug-resistant strains have made timely and accurate diagnosis and effective treatment an urgent problem worldwide.

Traditional methods of TB diagnosis mainly rely on microscopic examination of sputum samples and pathogenetic culture techniques. However, these methods have many limitations. First, microscopic examination, although quick, is less sensitive, especially in patients with low levels of MTB in sputum, and this method is difficult to detect early infection. In addition, sputum culture, although highly specific, has a culture time of several weeks, which does not meet the need for rapid diagnosis. With the progress of modern molecular biology technology, PCR-based nucleic acid amplification technology provides a more effective method for the early diagnosis of tuberculosis, especially in the case of limited sputum samples, PCR can rapidly amplify the pathogen DNA, which greatly improves the detection sensitivity. However, even so, PCR technology still has certain limitations, such as for patients with little or no sputum, sputum samples are difficult to obtain, making it difficult to carry out PCR testing [3,4]. During the past few years, considerable scientific efforts have been directed toward investigating blood-derived diagnostic methods for tuberculosis, particularly through the detection of biomarkers of host immune response, with a view to discovering alternative diagnostic tools to non-sputum specimens. Blood samples are easier to obtain and reflect the systemic immune response in the patient. Blood gene expression studies have shown that TB infection causes up- or down-regulation of a wide range of immune-related genes in the host, opening the possibility of developing gene expression-based diagnostic tools [5,6]. In the pathogenesis of MTB infection, adhesion involving pathogens to alveolar macrophages and dendritic cells (DCs) is the initial and crucial stage. In order to effectively destroy the pathogen, macrophages must recognize, phagocytose and clear *Mycobacterium tuberculosis* [7]. Members of the PTP family play a fundamental role in orchestrating diverse signaling pathways, and the O-type protein tyrosine phosphatase receptor (PTPRO), a member of the PTP family, belongs to the R3 subfamily of receptor-like protein tyrosine phosphatases, which are widely expressed in tissues such as the lungs, the heart, the kidneys, and the brain, and are involved in a number of biological processes such as intracellular signaling, cell growth and differentiation [8,9]. It has been found that macrophages in the liver form a negative feedback mechanism through PTPRO, a pro-inflammatory protein, which reduces reactive oxygen species (ROS) production by promoting mitochondrial autophagy, thereby limiting the inflammatory response. Therefore, protecting macrophages from the damaging effects of PTPRO is essential for maintaining the stability of the microenvironment [10]. In recent years, the study of PTPRO as a tumor suppressor gene has gradually increased, and its oncogenic role in lung cancer in particular has been confirmed by a number of studies [11]. By regulating phosphorylation and modulating diverse signaling pathways, PTPRO suppresses the proliferation and migration of tumor cells. TLR4 functions as a critical receptor in the immune response to tuberculosis, with its expression on macrophages allowing recognition of *Mycobacterium tuberculosis* [12]. TLR4 activates NF-κB through a MyD88-dependent pathway, leading to the expression of inflammatory factors [13]. NF-κB is an important signaling cascade that regulates the inflammatory response of host cells [14]. Studies have shown that defects in the PTPRO gene in macrophages impede the activation of TLR4 and its associated NF-κB signaling pathway, which in turn reduces the production of pro-inflammatory cytokines. Comparatively, overexpression of PTPRO not only enhances the function of TLR4, but also exacerbates the macrophage-induced inflammatory response, which may exacerbate local tissue damage and organ dysfunction [15].

PTPRO has been shown to be essential for inflammatory response and immune regulation, suggesting its potential role in tuberculosis [9,10,15]. But the involvement of PTPRO in tuberculosis has been scarcely explored, and no study has systematically elucidated the specific functions of PTPRO-related genes in TB. The host immune response is activated through multiple sophisticated mechanisms after MTB infection in which macrophages play a key role in the process of pathogen clearance. As the first line of defense of host innate immunity, macrophages are not only able to phagocytose and kill *Mycobacterium tuberculosis*, but also activate subsequent T-cell immune responses through antigen presentation [16]. Changes in PTPRO expression in macrophages may directly affect these immune response processes, Hence, exploring the functions of PTPRO and its relevant differentially expressed genes in tuberculosis is necessary.

In this study, we integrated public transcriptomic datasets with preliminary experimental validation to identify a PTPRO-related transcriptional signature associated with tuberculosis. TCGA-LUSC was used as a large human transcriptomic resource to screen genes correlated with PTPRO expression, while TB-related whole-blood datasets were subsequently used to refine this candidate gene set. Specifically, genes retained in the final signature were required to show TB-relevant expression patterns in comparisons with healthy controls and selected respiratory disease groups. We then assessed the ability of the resulting signature to distinguish TB from non-TB groups and examined its changes during anti-tuberculosis treatment. Because the clinical RT-qPCR cohort was small and exploratory, the present study was not intended to establish a clinically validated biomarker, but rather to provide preliminary evidence for a candidate blood transcriptional signature that merits further investigation in larger prospective cohorts and mechanistic studies.

Previous studies have reported several blood transcriptional signatures for TB diagnosis, progression risk, and treatment monitoring, including the BATF2/GBP5/SCARF1 signature, RISK11, and other multigene classifiers. The PO5 signature proposed here should therefore be regarded as a complementary, hypothesis-generating PTPRO-related gene panel, rather than a replacement for previously established TB transcriptomic signatures.

## 2. Materials and Methods

### 2.1. THP-1 Cell Culture and H37Ra Exposure

THP-1 cells (Guangzhou Saiku Biotechnology Co., Ltd., Guangzhou, China) were cultured in RPMI 1640 medium (ATCC, Manassas, VA, USA) supplemented with 10% fetal bovine serum and 1% penicillin-streptomycin according to the manufacturer’s instructions. When the cells reached approximately 1 × 10^6^ cells/mL, phorbol 12-myristate 13-acetate (PMA, 100 ng/mL) was added for 24 h to induce macrophage-like differentiation [17]. Differentiation was assessed by microscopic observation of cell adhesion and pseudopod formation and by flow cytometric analysis after staining with a FITC-conjugated anti-CD11b monoclonal antibody. To establish an in vitro mycobacterial exposure model, THP-1-derived macrophage-like cells were exposed to the attenuated *Mycobacterium tuberculosis* strain H37Ra (Chinese Medical Bacteria Preservation and Management Center, Beijing, China) at a multiplicity of infection of 10:1 for 24 h. H37Ra was selected as an attenuated strain for a preliminary and experimentally accessible model. Therefore, this model was intended to provide supportive evidence for infection-associated transcriptional changes and was not designed to fully recapitulate host responses induced by virulent laboratory strains or clinical isolates.

### 2.2. RT-qPCR

Total RNA was isolated from H37Ra-infected and control THP-1 cells using the Trizol reagent, according to the manufacturer’s protocol. RNA concentration and purity were assessed using a spectrophotometer. Complementary DNA (cDNA) was then synthesized with the Easy Script One-Step gDNA Removal & cDNA Synthesis SuperMix (AllStyle Gold Biotechnology, Beijing, China). Real-time quantitative PCR (RT-qPCR) was carried out using PerfectStart Green qPCR SuperMix (AllStyle), with GAPDH serving as the internal reference. Relative gene expression levels were determined using the 2^−ΔΔCt^ method. Primer sequences used for RT-qPCR are listed in Supplementary Table S1.

### 2.3. Transcriptomic Datasets

RNA-sequencing data and corresponding clinical information for lung squamous cell carcinoma were obtained from The Cancer Genome Atlas Lung Squamous Cell Carcinoma project (TCGA-LUSC; https://portal.gdc.cancer.gov/projects/TCGA-LUSC, accessed on 20 January 2025). In this study, TCGA-LUSC was used as a human transcriptomic resource to screen genes correlated with PTPRO expression, rather than as a tuberculosis cohort. After data preprocessing, samples with available gene-expression profiles and valid PTPRO expression values were included in the analysis.

Gene selection was carried out in two steps. First, TCGA-LUSC samples were divided into PTPRO-High and PTPRO-Low groups according to the median PTPRO expression level. Differentially expressed genes (DEGs) between the two groups were identified using the limma R package. Genes with a false discovery rate (FDR) < 0.05 and |log2 fold change| > 1 were selected as PTPRO-correlated candidate genes. Second, these candidate genes were further screened in the whole-blood GEO dataset GSE42830, which includes healthy controls, patients with active tuberculosis, patients with pneumonia, and patients with lung cancer. To identify genes more relevant to the blood transcriptional profile of TB, we retained genes that were upregulated in TB compared with healthy controls, pneumonia, and lung cancer. The overlapping genes between the TCGA-LUSC PTPRO-correlated gene set and the TB-related upregulated gene sets from GSE42830 were defined as the PTPRO-related five-gene signature, termed PO5.

This approach allowed us to first obtain a broad PTPRO-correlated gene set and then refine it in a TB-related blood transcriptomic context. Therefore, PO5 was considered a PTPRO-related candidate blood transcriptional signature associated with TB, rather than direct evidence of TB-specific or causally PTPRO-regulated genes. The second filtering step was used to reduce the influence of nonspecific inflammatory responses and tumor-associated transcriptional changes, although such effects could not be fully excluded.

Three independent GEO datasets, GSE19491, GSE42826, and GSE54992, were used as validation cohorts to assess the discriminatory performance of PO5 and its changes during anti-tuberculosis treatment. All analyses were performed using R software version 4.3.1. DEG results were visualized using the ggplot2 and pheatmap packages.

### 2.4. Tuberculosis Risk Score


$$S=\sum_{i=1}^{5}\frac{e_{i}-\mu_{i}}{\tau_{i}}$$

A TB risk score was assigned to each subject based on previously published gene-signature scoring approaches [18,19]. For each subject, the risk score was calculated from the normalized expression values of the five genes included in the PO5 signature. In this calculation, the expression value of each gene was standardized using the corresponding mean (μ_i_) and standard deviation (τ_i_) across the analyzed cohort, where μ_i_ represents the mean expression level of gene *i* and τ_i_ represents the standard deviation of gene *i*. A higher TB risk score indicates a greater likelihood of TB.

### 2.5. Study Participants and Ethics Statement

This exploratory clinical study was conducted in accordance with the Declaration of Helsinki and was approved by the Ethics Committee of Bengbu Medical University (Approval No. 514-2025). Participants were recruited by The First Affiliated Hospital of Bengbu Medical University and included individuals with active TB (*n* = 3), post-treatment TB (*n* = 3), pneumonia (*n* = 3), and healthy controls (HC, *n* = 3). The comparison of baseline data (mean, M (IQR)) for the four groups of participants is shown in Supplementary Table S2. Written informed consent was obtained from all participants prior to enrollment. All participants were selected based on their medical histories, questionnaire responses, and general physical examinations. Active TB infection was confirmed in TB participants through bacteriological examination (at least one positive result from sputum smear, *Mycobacterium tuberculosis* culture, or nucleic acid amplification test). These patients had been newly diagnosed with TB and had received anti-tuberculosis treatment for less than 7 days. Post-treatment TB samples were obtained from tuberculosis patients who had completed a course of anti-tuberculosis drug treatment. HC had no history of contact with or exposure to *Mycobacterium tuberculosis*, no recent history of infectious diseases, and normal chest X-ray results upon physical examination. All participants tested negative for human immunodeficiency virus (HIV). Because only three samples were included per clinical group, this cohort was considered a small exploratory validation cohort and was not intended to provide definitive evidence of clinical diagnostic performance.

### 2.6. Peripheral Blood Mononuclear Cell (PBMC) Preparation

PBMC isolation was performed as previously described, with minor modifications [20,21]. Briefly, peripheral blood samples were collected and mixed with an equal volume of sterile phosphate-buffered saline (PBS). The mixture was layered over Ficoll-Paque (GE Healthcare, Chicago, IL, USA) and centrifuged at 400 g for 30 min at 22 °C. The mononuclear cell layer was carefully aspirated, washed twice with PBS, and resuspended in RPMI-1640 medium supplemented with 10% FBS and 1% penicillin-streptomycin. The final concentration was adjusted to 1 × 10^6^ cells/mL for subsequent applications. Given the limited size of the clinical RT-qPCR cohort, statistical results from these samples were interpreted cautiously and considered exploratory.

### 2.7. Assessment of PTPRO Expression in Public TB Data and H37Ra-Exposed Macrophages

PTPRO expression was evaluated in the GSE42830 whole-blood dataset and in H37Ra-exposed THP-1-derived macrophage-like cells. In GSE42830, PTPRO expression was compared between healthy controls and active TB patients. In vitro, PTPRO mRNA expression was measured by RT-qPCR after 24 h of H37Ra exposure. This analysis was designed to assess the association between PTPRO expression and TB-related host responses, rather than TB incidence.

### 2.8. Statistical Analysis

All data analyses, unless otherwise specified, were conducted using R software (version 4.3.1). Real-time quantitative PCR (RT-qPCR) results were analyzed separately using SPSS software (version 29). To visualize the variance within the high-dimensional gene expression data, principal component analysis (PCA) was performed using the ‘principal component analysis’ package in R. Statistical comparisons were made using unpaired Student’s *t*-tests or analysis of variance (ANOVA) as appropriate for the experimental design. Where ANOVA was employed, post-hoc analysis was performed using Tukey’s multiple comparisons test. Statistical significance was defined as a *p*-value of less than 0.05. Statistical annotations are shown in the figures; however, results from the exploratory clinical RT-qPCR cohort should be interpreted cautiously because of the small sample size.

## 3. Results

### 3.1. PTPRO Expression Is Elevated in TB Whole-Blood Data and in H37Ra-Infected Macrophages

**Figure 1:**
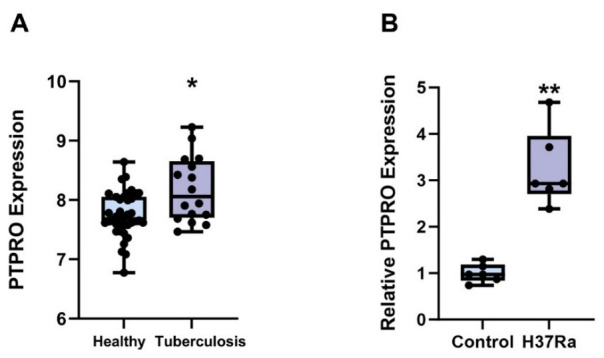
PTPRO expression in peripheral blood transcriptomic data from patients with active tuberculosis and in H37Ra-infected THP-1-derived macrophages. (**A**) Scatter-box plots show the log2-transformed expression levels of PTPRO in the full eligible healthy control and TB samples from GSE42830. Each dot represents one sample. (**B**) PTPRO mRNA expression in THP-1-derived macrophages after H37Ra infection (MOI = 10:1, 24 h). Compared with the control group, PTPRO expression was increased in H37Ra-infected cells. * *p* < 0.05 compared with healthy controls; ** *p* < 0.01 compared with the control group.

PTPRO expression levels were analyzed in the GSE42830 whole-blood gene expression dataset using the full eligible healthy control and TB samples. Differential expression analysis revealed that PTPRO mRNA expression was significantly increased in TB patients compared with healthy controls, indicating elevated PTPRO expression in tuberculosis (Figure 1A).

THP-1 cells were differentiated into macrophages and then challenged with *Mycobacterium tuberculosis* H37Ra at an MOI of 10:1. Successful infection was confirmed by observing bacterial adherence-associated morphological changes, pseudopod formation, and increased CD11b abundance. Twenty-four hours after infection, RNA was extracted from both infected and uninfected control cells and analyzed by RT-qPCR. The results demonstrated that PTPRO mRNA expression was significantly upregulated in H37Ra-infected THP-1-derived macrophages compared with controls (Figure 1B), suggesting that PTPRO expression is responsive to mycobacterial infection in this preliminary in vitro model.

Taken together, the public-dataset analysis and the in vitro findings support a potential association between PTPRO expression and TB-related host responses.

### 3.2. Identification of a PTPRO-Related Five-Gene (PO5) Signature in TB Through a Two-Step Screening Strategy

**Figure 2:**
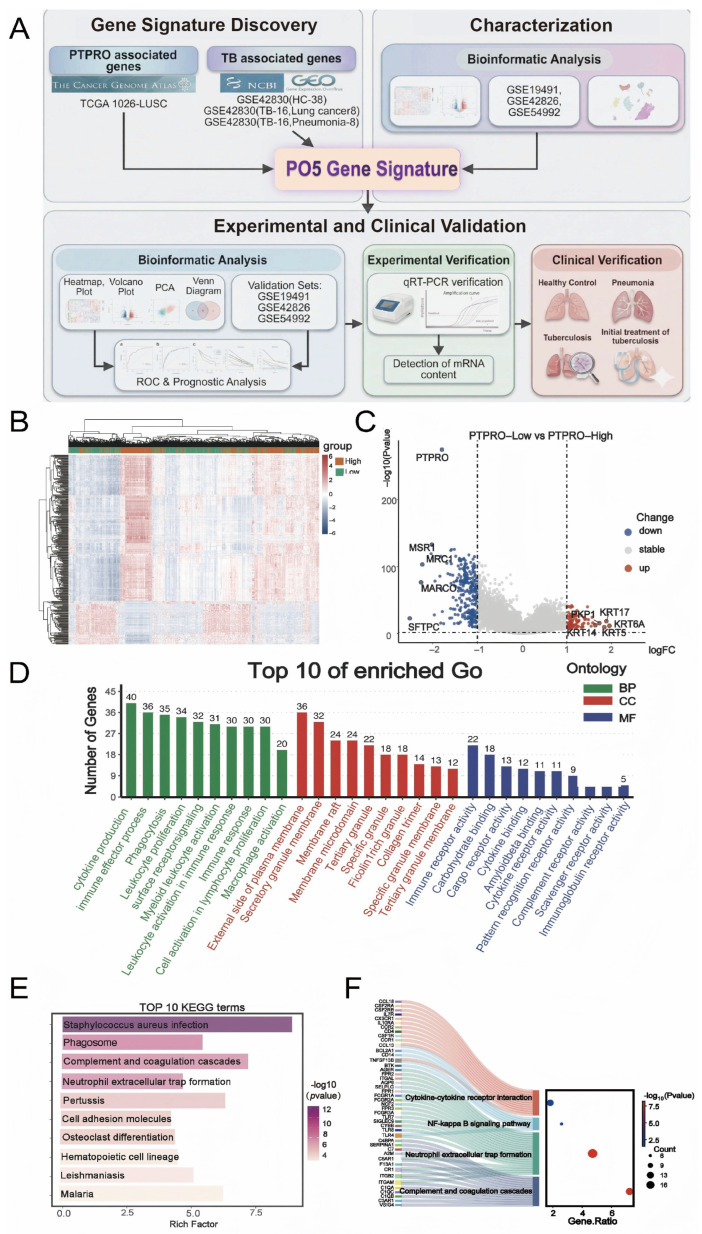
Two-step identification workflow and functional annotation of PTPRO-correlated candidate genes from the TCGA-LUSC discovery resource. (**A**) Workflow for the identification and validation of the PTPRO-related five-gene signature (PO5). The gene-selection strategy was performed in two sequential screening steps. In Step 1, the TCGA-LUSC cohort was used as a high-sample-size human transcriptomic discovery resource to identify genes correlated with PTPRO expression. Samples were stratified into PTPRO-High and PTPRO-Low groups according to the median PTPRO expression level, and differentially expressed genes (DEGs) between the two groups were identified. This step was not intended to imply that lung cancer and tuberculosis share identical transcriptional programs, but only to generate an initial PTPRO-correlated candidate gene set. In Step 2, the candidate genes from Step 1 were filtered using the independent whole-blood GEO discovery dataset GSE42830 by intersecting genes upregulated in TB vs. healthy controls, TB vs. pneumonia, and TB vs. lung cancer. Genes retained after this second filtering step were considered both PTPRO-related and TB-relevant within the available blood transcriptomic data, yielding the final five-gene signature, PO5. The PO5 signature was subsequently evaluated in independent GEO validation cohorts and explored by RT-qPCR in H37Ra-infected THP-1-derived macrophages and in a small exploratory clinical blood cohort. (**B**) Heatmap showing differentially expressed genes between PTPRO-High and PTPRO-Low groups in the TCGA-LUSC discovery resource. (**C**) Volcano plot showing upregulated and downregulated genes associated with PTPRO expression. (**D**) Gene Ontology (GO) enrichment analysis of PTPRO-correlated candidate genes, including biological process (BP), cellular component (CC), and molecular function (MF) categories. (**E**,**F**) KEGG pathway enrichment analysis of PTPRO-correlated candidate genes. The enrichment results indicate that these genes are involved in immune-related biological processes and pathways, supporting their further evaluation in a TB-relevant blood transcriptomic context. DEG thresholds were FDR < 0.05 and |log2 fold change| > 1.

We next identified a PTPRO-related candidate gene signature associated with tuberculosis using a two-step screening strategy (Figure 2A). Importantly, we did not assume that lung cancer and tuberculosis share identical transcriptional programs. Instead, the TCGA-LUSC dataset was used only as a high-sample-size transcriptomic discovery resource to identify genes whose expression co-varied with PTPRO, whereas TB whole-blood transcriptomic data were used in a second step to restrict the candidate gene set to genes showing TB-relevant expression patterns. In Step 1, we analyzed RNA-sequencing data from eligible TCGA-LUSC samples with available gene-expression profiles and valid PTPRO expression values. Samples were divided into PTPRO-High and PTPRO-Low groups according to the median expression level of PTPRO. Differential expression analysis between the two groups identified 80 upregulated genes and 349 downregulated genes (FDR < 0.05 and |log2FC| > 1). These genes were regarded as an initial PTPRO-correlated candidate gene set and are shown in the heatmap (Figure 2B) and volcano plot (Figure 2C). Functional enrichment analysis further indicated that these genes were enriched in immune-related biological processes and pathways (Figure 2D–F). Functional enrichment analysis was performed using GO and KEGG annotations [22,23].

In Step 2, we applied TB-oriented filtering using the whole-blood GEO discovery dataset GSE42830, which includes healthy controls, pneumonia, lung cancer, and TB samples. We first identified genes significantly upregulated in TB vs. healthy controls, yielding a TB-related gene set. To further reduce the influence of disease-nonspecific inflammation and potential tumor-related transcriptional confounding, we then restricted this set by additionally requiring upregulation in TB vs. pneumonia and TB vs. lung cancer. Using this sequential filtering strategy, we observed that a subset of genes from the PTPRO-correlated candidate pool identified in Step 1 also showed consistent TB-relevant upregulation in blood transcriptomic comparisons. We acknowledge that some of these overlapping genes may still reflect broader inflammatory or stress-associated host responses rather than TB-specific PTPRO biology. Therefore, the purpose of this step was not to establish TB-specific mechanistic regulation by PTPRO, but rather to enrich for genes that were both PTPRO-related and TB-relevant within the available datasets.

To further assess whether the PTPRO-correlated candidate genes identified in Step 1 were also relevant in a TB blood transcriptomic context, we analyzed the whole-blood dataset GSE42830, which includes healthy controls, pneumonia patients, lung cancer patients, and TB patients. Differential expression analysis comparing TB with healthy controls identified genes significantly upregulated in TB. A subset of these genes overlapped with the PTPRO-correlated candidate genes identified from TCGA-LUSC. Additional comparisons between TB and lung cancer and between TB and pneumonia were then used as a disease-context restriction step to reduce nonspecific inflammatory and tumor-related confounding.

**Figure 3:**
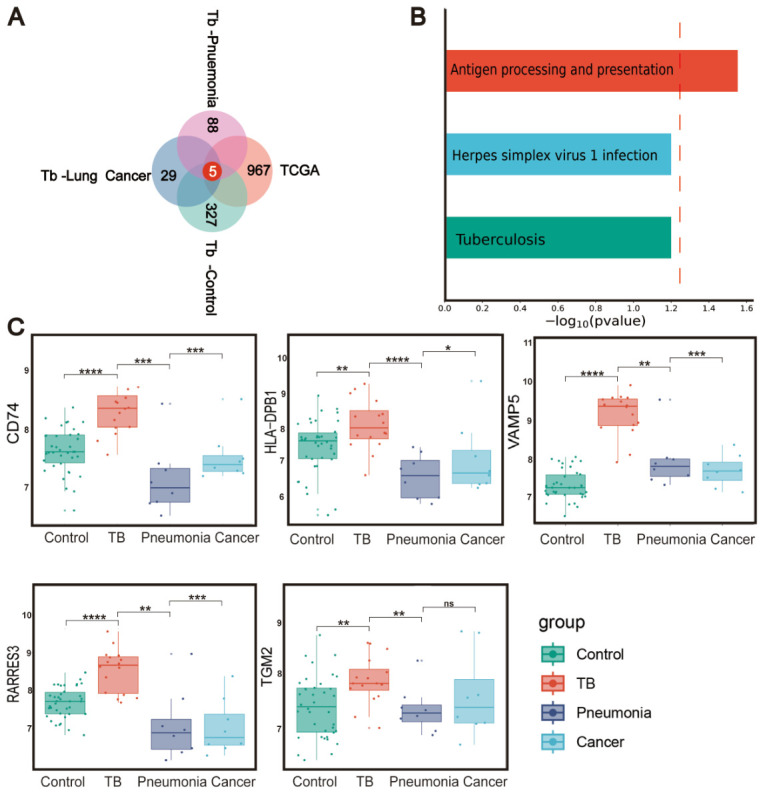
PO5 gene signature. (**A**) Venn diagram of the four differential gene sets, identifying five shared differentially expressed genes. (**B**) The KEGG pathway terms associated with the PO5 gene signature. The *p*-values were calculated by Fisher’s exact test. The dash line denotes the significance level of 0.05. (**C**) The expression levels of the PO5 gene signature in healthy controls, pneumonia patients, lung cancer patients, and TB patients. *, *p* < 0.05; **, *p* < 0.01; ***, *p* < 0.001; ****, *p* < 0.0001; ns, not significant, compared to TB group.

Although these overlapping genes may still include transcripts reflecting broader inflammatory or stress-associated host responses, this filtering strategy enriched for genes that were both PTPRO-related and TB-relevant within the available datasets. Finally, by intersecting genes upregulated in (1) PTPRO-High vs. PTPRO-Low TCGA-LUSC, (2) TB vs. healthy controls, (3) TB vs. lung cancer, and (4) TB vs. pneumonia (Figure 3A), we identified a core set of five shared genes (Supplementary Table S3): VAMP5, CD74, HLA-DPB1, RARRES3, and TGM2. These genes were enriched in the antigen processing and presentation pathway (Figure 3B) and were consistently upregulated in TB patients compared with healthy controls, pneumonia patients, and lung cancer patients (Figure 3C). We therefore defined these five genes as the PTPRO-related five-gene signature (PO5). Given the design of the screening framework, this signature should be interpreted as a PTPRO-related/PTPRO-correlated candidate signature associated with TB, rather than as direct evidence that these genes are tuberculosis-specific or causally regulated by PTPRO.

### 3.3. The Expression of PO5 Genes Was Elevated in Macrophages Infected with H37Ra

**Figure 4:**
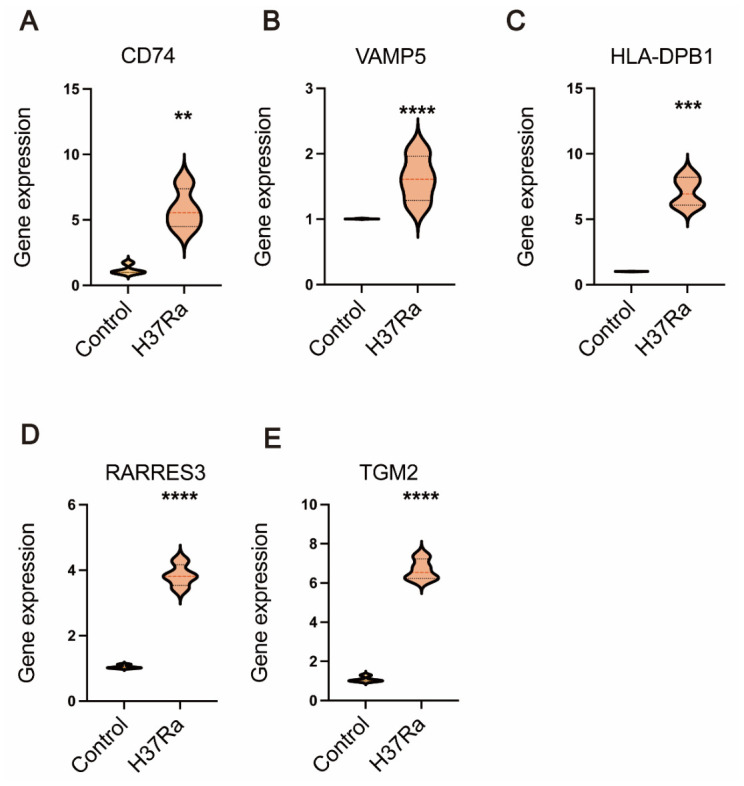
mRNA expression levels of five gene signatures in macrophages infected with H37Ra. Following the induction of THP-1 cells into macrophages using PMA, the cells were subsequently infected with *MTB* H37Ra strain (MOI = 10:1). The mRNA expression levels of CD74 (**A**), VAMP5 (**B**), HLA-DPB1 (**C**), RARRES3 (**D**) and TGM2 (**E**) were evaluated. **, *p* < 0.01; ***, *p* < 0.001; ****, *p* < 0.0001 compared to control group.

To provide preliminary experimental support, we next examined whether the PO5 genes were upregulated in macrophages following mycobacterial infection. Following PMA-induced differentiation into macrophages for 24 h, THP-1 cells were infected with *Mycobacterium tuberculosis* H37Ra (MOI = 10:1) for 24 h. In contrast, control macrophages remained uninfected. RT-qPCR analysis demonstrated a significant increase in the mRNA expression of HLA-DPB1, RARRES3, TGM2, VAMP5, and CD74 in H37Ra-infected macrophages compared with uninfected controls (Figure 4). These findings provide preliminary experimental support that the PO5 genes are infection-responsive in a mycobacterial infection-relevant in vitro model. However, because this experiment was performed only in an H37Ra-based cell model, these results should be interpreted as supportive rather than definitive evidence for broader clinical or strain-independent applicability.

### 3.4. PO5 Signature Shows Potential for Distinguishing TB from Healthy Controls, Latent TB, Pneumonia, and Lung Cancer in Independent Validation Cohorts

**Figure 5:**
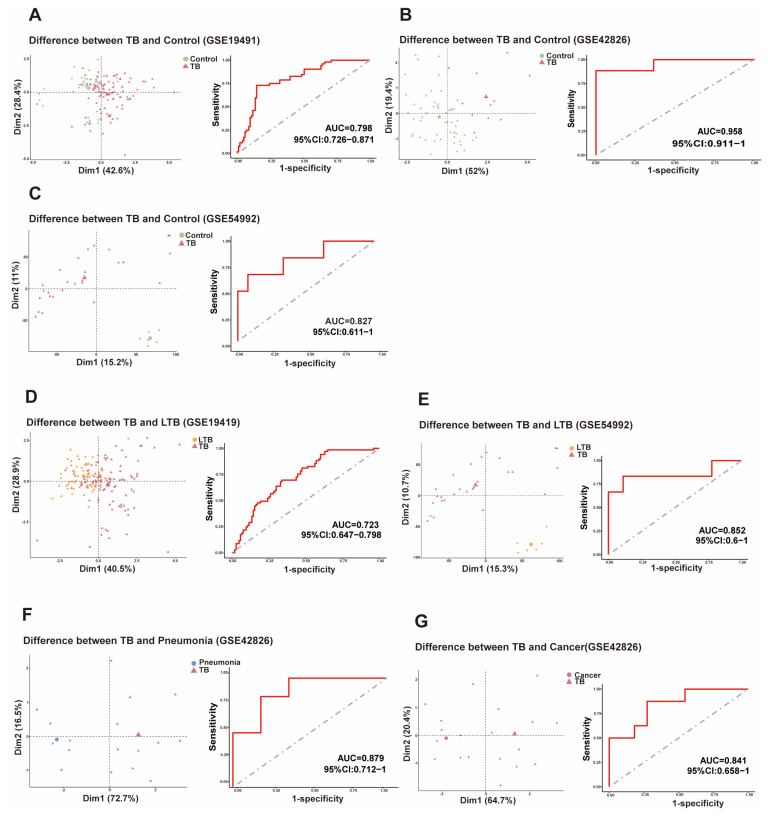
Evaluation of the discriminatory performance of the PO5 gene signature in public validation cohorts. The discriminatory performance and reliability of the PO5 gene signature for distinguishing TB patients from non-TB groups were assessed through principal component analysis (PCA) and ROC curve analysis. The signature’s ability to differentiate TB from healthy controls (**A**–**C**), latent TB (LTB) (**D**,**E**), pneumonia (**F**) and lung cancer (**G**) was assessed across multiple cohorts. (**A**–**C**) PCA and ROC analyses of the PO5 gene signature distinguishing TB from healthy controls in cohorts GSE19491, GSE42826, and GSE54992. (**D**,**E**) PCA and ROC analyses of the PO5 gene signature differentiating TB from LTB in cohorts GSE19491 and GSE54992. (**F**) PCA and ROC analyses of the PO5 gene signature differentiating TB from pneumonia in cohort GSE42826. (**G**) PCA and ROC analyses of the PO5 gene signature distinguishing TB from lung cancer in cohort GSE42826. (**Left**) panels: PCA plots based on the PO5 gene signature, with Dim1 (Principal Component 1) and Dim2 (Principal Component 2) representing the axes of maximum variance. In the PCA plots, the large legend represents the group average, and the distance between the large legends represents the size of the difference between the groups. (**Right**) panels: ROC curves evaluating sensitivity and specificity, with the area under the curve (AUC) calculated to assess classification performance.

We next evaluated the discriminatory performance of the PO5 signature in three independent GEO validation cohorts (GSE19491, GSE42826, and GSE54992). A TB risk score was calculated for each subject as a combined measure of the five-gene signature, with higher scores indicating a greater likelihood of TB. Across validation cohorts, TB patients showed higher TB risk scores than healthy controls, and receiver operating characteristic (ROC) analyses suggested that the PO5 signature has the potential to distinguish TB from healthy controls, latent TB, pneumonia, and lung cancer (Figure 5). These findings support the potential utility of the PO5 signature in multiple diagnostic comparison settings across public datasets.

We further assessed the classification ability of the 5-gene signature in distinguishing TB from latent tuberculosis (LTB) in two validation cohorts. TB patients had significantly higher TB scores based on the PO5 gene signature compared to LTB patients (Figure 5D,E). The area under the receiver operating characteristic curve (AUC) was 0.723 and 0.852 for GSE19491 and GSE54992, respectively.

We further evaluated the PO5’s ability to differentiate TB from other respiratory conditions. Analysis of the GSE42826 dataset revealed that TB patients exhibited significantly elevated TB risk scores compared with pneumonia patients (AUC = 0.879; Figure 5F,G). Similarly, when distinguishing TB from lung cancer within the same dataset, TB patients showed significantly elevated TB risk scores (AUC = 0.841; Figure 5G). These results support the potential discriminatory utility of the signature in public validation datasets.

### 3.5. PO5-Derived TB Risk Scores Track Anti-Tuberculosis Treatment Response

**Figure 6:**
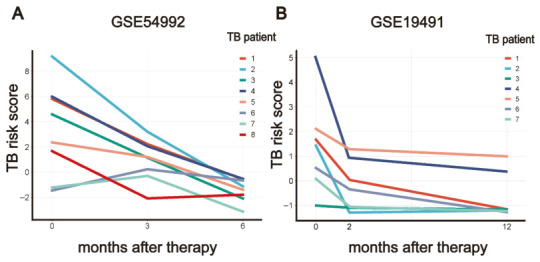
Changes in PO5-derived TB risk scores during anti-tuberculosis treatment. TB risk scores derived from PO5 gene signature were evaluated in patients with anti-TB treatment prognosis data from cohorts GSE54992 and GSE19491. (**A**) In cohort GSE54992, TB patients receiving 6-month anti-TB treatment showed significantly lower TB scores compared to their pre-treatment scores. (**B**) In cohort GSE19491, TB patients receiving 12-month anti-TB treatment exhibited significantly lower TB scores compared to their pre-treatment scores.

To investigate the PO5’s correlation with anti-tuberculosis treatment, we analyzed data from GSE54992 (*n* = 8 treated TB patients) and GSE19491 (*n* = 7 treated TB patients). Our analysis revealed a consistent trend of PO5-derived TB risk scores after the initiation of anti-tuberculosis chemotherapy in both datasets. In GSE54992 (Figure 6A), TB risk scores showed a progressive decline with treatment duration, being lowest in patients treated for 6 months, intermediate in those treated for 3 months, and highest in untreated patients. Similarly, in GSE19491 (Figure 6B), TB risk scores were lower after 2 months of treatment compared to untreated patients and further decreased after 12 months. These results from two independent validation cohorts indicate that the PO5-derived TB risk score has the potential to monitor the effectiveness of anti-TB treatment.

### 3.6. Exploratory Clinical RT-qPCR Assessment of PO5 Genes in HC, Pneumonia, TB, and Post-Treatment TB

**Figure 7:**
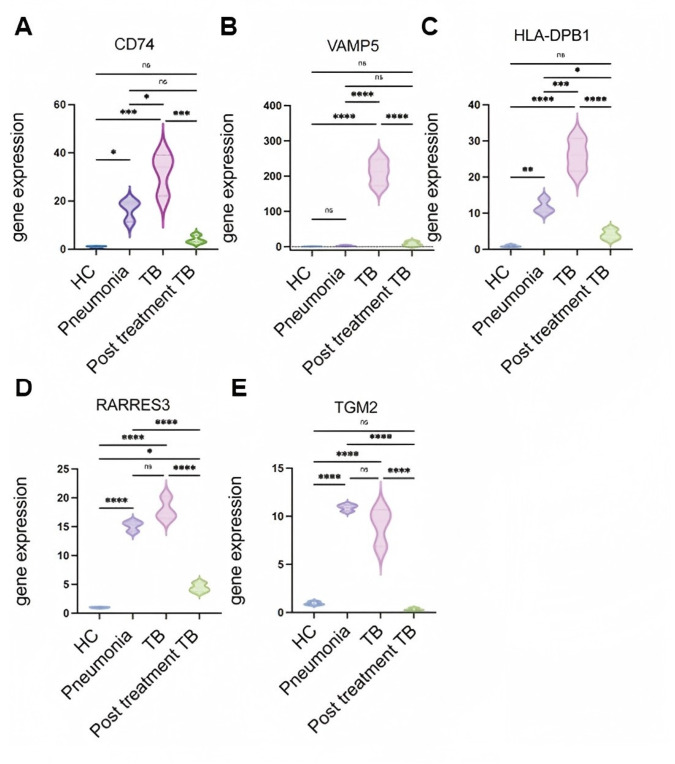
mRNA Expression Levels of PO5 genes in HC, Pneumonia, TB, and Post-Treatment TB. Blood samples from four participant groups were analyzed for mRNA expression changes of CD74 (**A**), VAMP5 (**B**), HLA-DPB1 (**C**), RARRES3 (**D**), and TGM2 (**E**) by RT-qPCR. Because each clinical group included only three samples, these data should be interpreted as exploratory. * *p* < 0.05; ** *p* < 0.01; *** *p* < 0.001; **** *p* < 0.0001 between the indicated groups; ns, not significant.

To further explore the potential relevance of PO5 in a clinical setting, we analyzed mRNA expression levels in blood samples from healthy controls (HC), pneumonia patients, active TB patients, and post-treatment TB patients (*n* = 3 per group) (Figure 7). RT-qPCR analysis showed that HLA-DPB1, VAMP5, RARRES3, and CD74 tended to be upregulated in active TB compared with some non-TB groups, and several of these genes showed reduced expression after treatment. In contrast, although TGM2 expression tended to be higher in TB than in pneumonia, no significant difference was observed in this small cohort.

Given the very small sample size, these clinical RT-qPCR data should be regarded as exploratory and should not be interpreted as evidence that any individual gene can robustly and independently distinguish TB from pneumonia or post-treatment TB. In particular, while TGM2 showed limited stand-alone discriminatory value in this cohort, it was retained in the PO5 signature because it emerged from the transcriptomic discovery pipeline and contributed to the performance of the multigene signature in the validation analyses. Consistent with this interpretation, additional analysis showed that a 4-gene model excluding TGM2 performed less well than the original 5-gene signature.

Overall, these findings support cautious interpretation of the clinical cohort as providing preliminary supportive evidence, while suggesting that the multigene signature context may be more informative and stable than the apparent performance of any single component gene in isolation.

## 4. Discussion

Protein tyrosine phosphatase receptor type O (PTPRO) is involved in multiple signaling pathways related to immune regulation, inflammation, and cellular differentiation [24,25,26,27]. Previous studies have linked PTPRO/PTPROt to inflammatory signaling and immune-cell function [9,10,11,27], suggesting potential relevance to TB host responses. In the present study, we observed elevated PTPRO expression in TB whole-blood transcriptomic data and in H37Ra-infected THP-1-derived macrophages, supporting a potential association between PTPRO expression and TB-related host responses.

Importantly, the PO5 signature was identified using a two-step screening framework, rather than by directly inferring TB biology from a lung cancer cohort. TCGA-LUSC was used only as a large transcriptomic discovery resource to identify genes whose expression co-varied with PTPRO, and this initial candidate pool was then restricted using independent TB whole-blood transcriptomic comparisons against healthy controls, pneumonia, and lung cancer. Thus, the final panel should be interpreted as a PTPRO-related/PTPRO-correlated candidate blood transcriptional signature associated with TB, rather than as proof of a conserved disease-specific PTPRO transcriptional network across lung cancer and tuberculosis.

The present findings should be interpreted alongside previous work on host blood transcriptional signatures for TB. Several such signatures have been proposed for TB diagnosis, progression-risk assessment, or treatment monitoring. Roe et al. described a three-gene signature consisting of BATF2, GBP5, and SCARF1 for short-term risk stratification among TB contacts [28]. Mulenga et al. examined the longitudinal behavior of the RISK11 blood transcriptomic signature in relation to TB risk and treatment-associated changes [29]. Vargas et al. reported a 45-gene model derived and validated across multiple cohorts for TB prognosis and treatment-response assessment [30]. Other studies have explored more clinically adaptable platforms and reduced gene panels, including the NanoString-based parsimonious signature developed by Kaipilyawar, Wang, and colleagues [31], and the six-gene RISK6 signature evaluated for TB diagnosis and treatment monitoring in multi-country cohorts [32]. Prospective multicentre studies have further shown that, although host blood transcriptomic biomarkers are promising, their performance may vary by clinical setting, symptom status, comparator disease group, and infection background [33,34]. In this context, PO5 should be viewed as a complementary PTPRO-related candidate signature, not as a replacement for existing TB blood signatures. Direct comparison with established signatures in larger prospective cohorts will be needed to define its added value.

This strategy has both strengths and limitations. It separates broad candidate discovery from disease-oriented restriction, but it does not demonstrate that the retained genes are tuberculosis-specific PTPRO-regulated targets. Some may still reflect more general inflammatory or stress-related host responses, and tumor-specific biology from the discovery cohort cannot be completely excluded. In addition, the present study does not establish a causal regulatory relationship between PTPRO and the five signature genes. Functional experiments, such as PTPRO knockdown or overexpression in *Mycobacterium tuberculosis*-infected macrophages, will be required to determine whether any of the five genes are directly downstream of PTPRO in the TB context.

The five PTPRO- related differential genes identified in this study include VAMP5, CD74, HLA-DPB1, RARRES3, and TGM2, whose significantly up-regulated expression in TB patients provides new possibilities for blood sample-based TB diagnosis. Traditional TB diagnostic methods (e.g., sputum testing) are often limited by the difficulty of sample acquisition and long testing time in clinical applications. Therefore, it is particularly important to develop rapid and accurate diagnostic methods based on blood samples. Our analyses across public datasets suggest that the PO5 signature has potential discriminatory utility for distinguishing TB from healthy controls and selected respiratory diseases. However, these findings should be interpreted as supportive evidence from retrospective transcriptomic datasets rather than as definitive proof of clinical performance.

**Figure 8:**
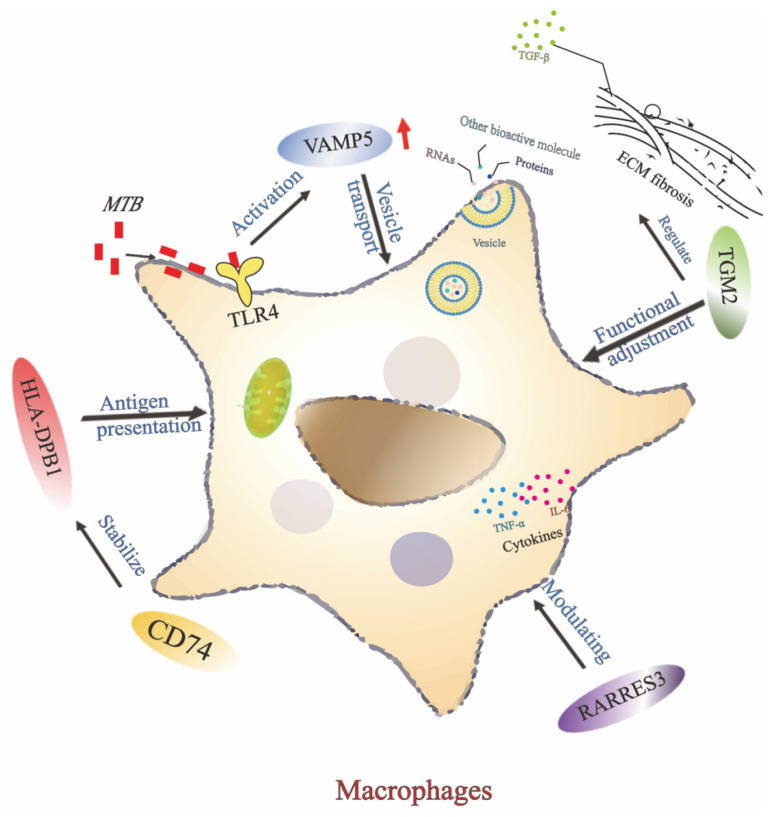
Summary of the potential roles of PO5 genes in macrophage responses to *Mycobacterium tuberculosis* infection. The roles of VAMP5, HLA-DPB1, CD74, RARRES3, and TGM2 in macrophage responses to MTB infection are depicted. MTB enters macrophages via surface receptors, activating signaling pathways (e.g., TLR4) that upregulate VAMP5, enhancing vesicle transport of MTB biomolecules (e.g., RNA) to phagosomes. Concurrently, macrophages engulf MTB and present bacterial peptides via MHC class II molecules, including HLA-DPB1, to CD4^+^ T cells, initiating an immune response. CD74 stabilizes and transports HLA-DPB1. RARRES3 modulates cytokine production (e.g., TNF-α and IL-6), influencing macrophage function, while TGM2 activates TGF-β1, promoting fibroblast activation and extracellular matrix (ECM) deposition, contributing to tuberculous granuloma formation to restrict MTB spread. Potential crosstalk among HLA-DPB1, CD74, RARRES3, TGM2, and VAMP5 regulates macrophage function and immune responses through shared signaling pathways, with HLA-DPB1 and CD74 central to antigen presentation, RARRES3 and TGM2 modulating inflammation, and VAMP5 facilitating intracellular trafficking. These interactions likely play a critical role in tuberculosis pathogenesis.

Interestingly, the five members of PO5 gene signature are closely related functionally. Among the five genes we screened, the VAMP5 gene is mainly involved in the vesicular transport process, which plays an critical role in antigen presentation and immune response in macrophages [35,36]. Macrophages, in recognizing and clearing *MTB*, need to deliver antigens to T cells via vesicular transport to initiate an adaptive immune response. The up-regulated expression of VAMP5 in patients with TB may be related to the resistance of host cells to pathogen invasion through enhanced antigen presentation. *MTB* releases biomolecules in phagosomes, such as RNA, lipopolysaccharides, lipoproteins, and other biomolecules, which are incorporated into the inclusion bodies. *MTB* infection induces the release of IFN-γ and activation of the TLR4 pathway, which increases the level of VAMP5 protein [37,38]. The CD74 gene encodes a chaperone protein for MHC-class II molecules, which plays a stabilizing role in antigen presentation. The up-regulated expression of CD74 suggests that the host immune system enhances antigen presentation in the face of *MTB* infection, resulting in an effective activation of the T-cell response [39,40,41,42]. These findings may suggest that CD74 contributes to the host-response signature observed in TB, although the current study does not support interpreting CD74 as a stand-alone diagnostic biomarker. Meanwhile, HLA-DPB1, an MHC class II cellular molecule, is involved in presenting exogenous antigens to CD4^+^ T cells [43]. In this process, polymorphisms in HLA-DPB1 may affect the function of CD74, which serves as a chaperone protein that contributes to the stabilization and transport of HLA-DPB1, and thus the immune response to *MTB* [44]. The present work revealed an upregulation in the expression of HLA-DPB1 in TB patients was closely associated with the activation of the host immune system. This finding is consistent with previous studies and further suggests that HLA-DPB1 may contribute significantly to the immune response to tuberculosis by enhancing the function of antigen presentation [45]. VAMP5 may enhance macrophage function by affecting the expression of CD74 and HLA-DPB1 and their transport. Whereas the role of macrophages in TB infection, especially in their ability to clear *MTB*, may be regulated by RARRES3 [44]. The up-regulated expression of RARRES3 in TB patients may suggest an enhanced antigen-presenting capacity by regulating cytokine production and macrophage function through differentiation and affecting the expression of HLA-DPB1 and CD74. These observations support the possible relevance of RARRES3 within the broader PO5 host-response signature, but not its use as an individual diagnostic marker based on the current data [46]. In addition, TGM2 is a multifunctional enzyme involved in the stabilization of the extracellular matrix and the regulation of apoptosis and inflammatory responses [47]. Up-regulated expression of TGM2 in tuberculosis may be associated with host cell inhibition of pathogen spread through the fibrotic response [48,49]. The fibrotic response contributes to the formation of tuberculosis granulomas, thus limiting the transmission of *MTB*, and this mechanism is therefore particularly important during chronic tuberculosis infection. TGM2 may participate in regulating macrophage function in conjunction with RARRES3 and CD74, affecting their immune response to *MTB* [50]. TGM2 can indirectly regulate HLA-DPB1 expression by affecting the polarization state of macrophages (Figure 8).

In addition to showing potential discriminatory value in public datasets, the PO5 signature may help distinguish TB from selected non-TB respiratory disease groups. In particular, the expression of CD74 and HLA-DPB1 was significantly lower in patients with lung cancer and pneumonia than in patients with TB, suggesting that these genes may contribute to the discrimination of TB from other lung diseases when considered as part of the multigene PO5 signature. This result is of great significance for clinical practice, especially when imaging tests cannot clearly differentiate between TB and lung cancer, gene expression-based tests can provide clinicians with more accurate auxiliary diagnostic tools. In our results, we found that the pneumonia group was not completely consistent in the expression of the five genes, which may be related to the different stages of the immune response and individual differences. Sometimes, in response to immune activation and inflammatory processes, their expression levels may be higher than in healthy individuals; at other times, with modulation of the immune response and remission of inflammation, their expression levels may decrease. Such dynamic changes are part of the body’s response to infection and may be affected by a combination of genetic and environmental factors. Dynamic changes in five gene markers during anti-tuberculosis treatment also provide a new perspective on treatment monitoring. Our study showed that the TB scores of patients gradually decreased with anti-tuberculosis treatment, especially after 6 months of treatment, and the gene expression levels decreased significantly. These findings suggest potential relevance of the signature to treatment-response assessment, although this interpretation remains exploratory and requires validation in larger prospective cohorts. The same expression trend was also observed in the clinical blood sample validation. Through the changes in gene expression, changes in gene expression may provide a basis for future studies evaluating molecular indicators of treatment response.

This treatment monitoring method based on the molecular level offers new possibilities for future TB treatment. While traditional methods of assessing treatment efficacy suffer from time delay and lack of precision, gene expression-based monitoring methods can reflect patients’ response to treatment more sensitively, thus providing strong support to support clinical decision-making.

Although this study validated the potential of five PTPRO- related differential genes in tuberculosis diagnosis and treatment monitoring through multiple sets of data, there are still some limitations. First, the limited sample size may affect the generalizability of the results, especially the applicability in different races, genders and age groups still needs further validation. Further investigations should include a larger sample and validate findings across multiple centers in different geographic and demographic contexts to ensure the global applicability of these gene markers. Secondly, this study is mainly based on the analysis of gene expression levels and has not yet delved into the specific biological functions of these genes in the pathogenesis of TB. Future studies could incorporate functional experiments (e.g., CRISPR gene editing technology) to further validate the underlying mechanism of action of these genes the underlying mechanism through which these genes act in the macrophage immune response. In addition, the development of multidimensional diagnostic models by combining genetic markers with other biomarkers (e.g., proteins, metabolites) may further enhance diagnostic accuracy and the sensitivity of monitoring treatment effects.

## 5. Conclusions

This study identified a PTPRO-related five-gene blood transcriptional signature, PO5, composed of VAMP5, CD74, HLA-DPB1, RARRES3, and TGM2. In public transcriptomic datasets, PO5 distinguished active TB from healthy controls, latent TB, pneumonia, and lung cancer, and the PO5-derived score decreased after anti-tuberculosis treatment. These findings suggest that PO5 may capture part of the blood transcriptional response associated with active TB and treatment-related changes.

The in vitro and clinical RT-qPCR results provided preliminary support for these observations, but their strength is limited by the use of the attenuated H37Ra strain and the small clinical sample size. Thus, PO5 should be regarded as an exploratory candidate signature rather than a validated clinical biomarker. Larger prospective studies and functional experiments are needed to confirm its diagnostic value, treatment-monitoring utility, and biological relationship with PTPRO-mediated host responses.

## Data Availability

Publicly available datasets were analyzed in this study. These datasets are available in the NCBI Gene Expression Omnibus (GEO) under accession numbers GSE42830, GSE19491, GSE42826, and GSE54992, and in The Cancer Genome Atlas (TCGA-LUSC) at the Genomic Data Commons portal. The original experimental data generated in this study, including RT-qPCR results and clinical validation data, are available from the corresponding authors upon request.

## Supplementary Materials

The following supporting information can be downloaded at: https://www.mdpi.com/article/10.3390/biomedicines14051021/s1: Table S1: RT-qPCR primer sequences for TGM2, RARRES3, HLA-DPB1, CD74, VAMP5, PTPRO, and GAPDH; Table S2: sociodemographic and clinical data of participants, including group, number of subjects, age, sex, and BMI; Table S3: functions and descriptions of the five-gene signature, including VAMP5, CD74, HLA-DPB1, RARRES3, and TGM2.

## Abbreviations

The following abbreviations are used in this manuscript:

AIDS	Acquired Immunodeficiency Syndrome
ANOVA	Analysis of Variance
AUC	Area Under the Curve
cDNA	Complementary DNA
DCs	Dendritic Cells
DEGs	Differentially Expressed Genes
ECM	Extracellular Matrix
FBS	Fetal Bovine Serum
FC	Fold Change
FDR	False Discovery Rate
FITC	Fluorescein Isothiocyanate
GAPDH	Glyceraldehyde-3-Phosphate Dehydrogenase
GEO	Gene Expression Omnibus
GO	Gene Ontology
HC	Healthy Controls
HIV	Human Immunodeficiency Virus
KEGG	Kyoto Encyclopedia of Genes and Genomes
LTB	Latent Tuberculosis
LUSC	Lung Squamous Cell Carcinoma
MDR	Multidrug-Resistant
MF	Molecular Function
MHC	Major Histocompatibility Complex
MOI	Multiplicity of Infection
MTB	*Mycobacterium tuberculosis*
MyD88	Myeloid Differentiation Primary Response 88
NF-κB	Nuclear Factor Kappa B
PBS	Phosphate-Buffered Saline
PCA	Principal Component Analysis
PBMCs	Peripheral Blood Mononuclear Cells
PCR	Polymerase Chain Reaction
PMA	Phorbol 12-Myristate 13-Acetate
PO5	PTPRO-Related Five-Gene Signature
PTPRO	Protein Tyrosine Phosphatase Receptor Type O
RARRES3	Retinoic Acid Receptor Responder 3
ROC	Receiver Operating Characteristic
ROS	Reactive Oxygen Species
RPMI	Roswell Park Memorial Institute
RT-qPCR	Real-Time Quantitative Polymerase Chain Reaction
TB	Tuberculosis
TCGA	The Cancer Genome Atlas
TGF-β	Transforming Growth Factor Beta
TGM2	Transglutaminase 2
TLR4	Toll-Like Receptor 4
TNF-α	Tumor Necrosis Factor Alpha
VAMP5	Vesicle-Associated Membrane Protein 5
WHO	World Health Organization
XDR	Extensively Drug-Resistant
