# Supplementary material for: A PTPRO-Related Five-Gene Blood Transcriptional Signature with Diagnostic Potential for Tuberculosis
Source: Biomedicines. 2026 Apr 30;14(5):1021. doi: 10.3390/biomedicines14051021 (PMC13203672; doi:10.3390/biomedicines14051021)
Supplement: Supplementary file 1 [file biomedicines-14-01021-s001.zip › biomedicines-4247340-supplementary.pdf]

Table S1. RT-qPCR Primer Sequence

Gene Name	5’-3’
TGM2-F	GAGAAGGAGGAGACAGGGATGG
TGM2-R	AGCGGTGTTGTTGGTGATGTG
RARRES3-F	GCTCCTCCAGTGTCTTCTCAGTC
RARRES3-R	ACAGCCTCCCACCACATCTTC
HLA-DPB1-F	GGATTTCTACCCAGGCAGCATTC
HLA-DPB1-R	TCCAGTCTCCATTACGGATCAGG
CD74-F	CATCCTGGTGACTCTGCTCCTC
CD74-R	AGGTTCTGGGAGGTGACTGTC
VAMP5-F	TCAACCTTCAACAAGACTACACAGAAC
VAMP5-R	AGCAGGACACCAACCACCAC
PTPRO-F	CCTTCTTCCTGCCACTGCCTAC
PTPRO-R	AGCACGGAGATCACTACCACATTG
GAPDH-F	GATTCCACCCATGGCAAATTC
GAPDH-R	CTGGAAGATGGTGATGGGATT

Table S2. Sociodemographic and clinical data of participants

	Group			
Date	HC	Pneumonia	TB	Post treatment TB
Number of subjects	3	3	3	3
Age(years)(m[IQR])	31 [11.5]	62 [12.0]	38 [22.0]	36 [17.5]
Sex (M: F)	2:1	2:1	2:1	1:2
BMI	22.4	24.6	21.8	23.6

Table S3. The functions and descriptions of five gene signatures

gene symbol	Gene function
VAMP5	A vesicle associated membrane protein involved in the transport and membrane fusion of intracellular vesicles. It plays a critical role in immune cells such as macrophages and T cells, affecting antigen presentation and immune response, especially in response to bacterial infections [24]
CD74	A companion protein of MHC class II molecules, mainly involved in the stability of antigen presentation. It plays a key role in regulating the function of macrophages and dendritic cells, promoting T cell activation and immune response [25]
HLA-DPB1	A component of the major histocompatibility complex (MHC) class II molecules, primarily involved in the antigen presentation process. It plays a pivotal role in the recognition and activation of T cells, capable of presenting exogenous antigens to $CD4^{+}$T cells, thereby initiating adaptive immune responses [26]
RARRES3	Related to cell differentiation and immune regulation. It plays a role in regulating the function of macrophages and promoting inflammatory responses, especially in enhancing immune responses against infections [27]
TGM2	A multifunctional enzyme involved in the stability of extracellular matrix, cell adhesion, and signal transduction. It plays a regulatory role in immune response, cell apoptosis, and inflammatory response, especially in chronic inflammation and fibrosis processes [28-30]
